# First Complete Genome Sequence of *Clostridium sporogenes* DSM 795^T^, a Nontoxigenic Surrogate for *Clostridium botulinum*, Determined Using PacBio Single-Molecule Real-Time Technology

**DOI:** 10.1128/genomeA.00832-15

**Published:** 2015-07-30

**Authors:** Kazuma Nakano, Yasunobu Terabayashi, Akino Shiroma, Makiko Shimoji, Hinako Tamotsu, Noriko Ashimine, Shun Ohki, Misuzu Shinzato, Kuniko Teruya, Kazuhito Satou, Takashi Hirano

**Affiliations:** Okinawa Institute of Advanced Sciences, Uruma, Okinawa, Japan

## Abstract

The first complete genome sequence of *Clostridium sporogenes* DSM 795^T^, a nontoxigenic surrogate for *Clostridium botulinum*, was determined in a single contig using the PacBio single-molecule real-time technology. The genome (4,142,990 bp; G+C content, 27.98%) included 86 sets of >1,000-bp identical sequence pairs and 380 tandem repeats.

## GENOME ANNOUNCEMENT

*Clostridium sporogenes* is an anaerobic spore-forming bacterium that causes food spoilage (1, 2). *C. sporogenes* is widely used as a nontoxigenic surrogate for *Clostridium botulinum* in the validation of food sterilization because of its physiological and phylogenetic similarity to *C. botulinum* and nontoxigenicity (2–6).

A draft sequence of *C. sporogenes* DSM 795^T^ has been determined using 454, Illumina, and Sanger technologies in 16 contigs (GenBank accession number JFBQ00000000) (total 4,106,665 bp; average G+C content, 27.8%) (A. Poehlein, R. Karin, S. M. Koenig, R. Daniel, and P. Duerre, submitted for publication) (7, 8). These contigs are disconnected at tandem repeat or low G+C regions. Here, we report the first complete genome sequence of *C. sporogenes* DSM 795^T^ determined using the PacBio single-molecule real-time (SMRT) technology (9).

The genomic DNA of *C. sporogenes* DSM 795^T^, originally isolated from soil in 1908, was obtained from the Deutsche Sammlung von Mikroorganismen und Zellkulturen (DSMZ) (10). It was purified using a PowerClean DNA cleanup kit (MoBio, Carlsbad, CA), followed by a 20-kb library construction for P5-C3 chemistry. After >7-kb size selection using BluePippin (Sage Science, Beverly, MA), 8 SMRT cells from the libraries were sequenced using the PacBio RS II platform (Pacific Biosciences, Menlo Park, CA) with 180-min movies. *De novo* assembly was performed using the hierarchical genome assembly process 2 (HGAP2) workflow (11). A single circular contig representing a chromosome was obtained (4,142,990 bp; average G+C content, 27.98%).

The complete genome sequence of *C. sporogenes* DSM 795^T^ included 86 sets of >1,000-bp identical sequence pairs (4,911-bp maximum) and 380 tandem repeats (369 bp × 8.5 copies maximum). Tandem repeats were identified using Tandem Repeats Finder (12). Recently, a sequence of *C. sporogenes* NCIMB 10696^T^, which originated from the same strain (McClung 2004^T^) as *C. sporogenes* DSM 795^T^, has been determined using 454, Illumina, and Sanger technologies (CP009225) (http://www.straininfo.net/strains/7982) (4,141,984 bp; average G+C content, 28.00%) (13). We found three marked differences between the sequences of DSM 795^T^ and NCIMB 10696^T^. First, in a 39-bp tandem region, DSM 795^T^ carried 25.5 copies (1,156,066 to 1,157,028), whereas 10696^T^ carried 20.5 copies (1,156,066 to 1,156,839). Second, in a 312-bp tandem region, DSM 795^T^ carried 5.9 copies (3,502,125 to 3,503,970), whereas 10696^T^ carried 4.9 copies (3,501,430 to 3,502,963). Third, DSM 795^T^ had a 501-bp extra region (2,040,199 to 2,040,699) that could be inserted in 10696^T^ (between 2,040,006 and 2,040,007). On DSM 795^T^ sequencing, the PacBio RS II platform produced extra-long reads with an average of 3,959 bp and a maximum of 35,904 bp, and large numbers of reads completely covered those regions: 290 reads for the first, 191 reads for the second, and 359 reads for the third. This result suggests that the number of tandem repeats is underestimated in the 10696^T^ sequence. The SMRT technology provides power for genome sequencing with multikilobase extra-long reads and unbiased G+C coverage (11, 14, 15) for assessing structural variations such as variable number tandem repeat.

### Nucleotide sequence accession number.

The complete genome sequence of *C. sporogenes* DSM 795^T^ was deposited in DDBJ/ENA/GenBank under the accession number CP011663.
